# Reply to Van Overmeire, R. Comment on “Tyson, G.; Wild, J. Post-Traumatic Stress Disorder Symptoms among Journalists Repeatedly Covering COVID-19 News. *Int. J. Environ. Res. Public Health* 2021, *18*, 8536”

**DOI:** 10.3390/ijerph182111423

**Published:** 2021-10-30

**Authors:** Gabriella Tyson, Jennifer Wild

**Affiliations:** Oxford Centre for Anxiety Disorders and Trauma, Department of Experimental Psychology, University of Oxford, Oxford OX1 1TW, UK; jennifer.wild@psy.ox.ac.uk

During the COVID-19 pandemic, journalists reporting on the crisis in the UK were classed as keyworkers. Their role was integral to keeping the public informed of adverse and often fatal consequences related to the spread of COVID-19 as well as government-mandated regulations. Tyson and Wild [1] report on the mental health of journalists working during the pandemic, finding that journalists who repeatedly covered COVID-19 news had greater PTSD symptoms than journalists working during the pandemic who did not repeatedly cover COVID-19. Van Overmeire [2] raised concerns about the validity of the findings, arguing that the research incorrectly classified criterion A events, thus widening the inclusion criteria for PTSD, and that the research potentially misinterpreted findings related to levels of PTSD symptoms and may even have ignored the statistical assumptions for the reported regression analysis. In this response letter, we elucidate the distinction between a diagnosis of PTSD and PTSD symptoms as well as how criterion A events were assessed, demonstrating that the research is aligned with the DSM-5 assessment of criterion A events. We clarify how the results demonstrate higher PTSD symptoms among journalists who repeatedly covered COVID-19. We also address Van Overmeire’s concerns related to the statistical assumptions of the analyses.

We first wish to thank Van Overmeire for his provocative comment. We welcome the opportunity for scientific debate and agree that clinical researchers should assess for criterion A trauma when investigating PTSD. Assessment of criterion A trauma ensures validity and leads to more accurately reported rates of the disorder. For example, we found in our recent study of healthcare workers that PTSD was most frequently associated with trauma that predated the current pandemic [3]. This is in contrast to other published studies, which relate high rates of PTSD to pandemic working, yet which fail to assess the trauma associated with symptoms. Our research demonstrated that assessment of criterion A trauma is essential to better understand presentations of mental ill health.

With reference to Tyson and Wild [1] and the diagnosis of PTSD, we respectfully re-iterate that we make no claims on diagnosing PTSD at any point in the report since we administered self-report questionnaires rather than structured clinical interviews. The reference to PTSD symptoms (rather than a diagnosis of PTSD) is in the title of the study, referenced throughout the paper, and is openly referred to as a limitation, where once again we state that the research assessed PTSD symptoms rather than a diagnosis. It is possible to experience PTSD symptoms without meeting a diagnosis of PTSD and, hence, clarity should be sought when reporting symptoms versus diagnoses. In more comprehensive studies of PTSD, we administer the Structured Clinical Interview for DSM-5 to assess for a PTSD diagnosis [4]. Measuring PTSD symptoms with the PCL-5 [5] is a commonly used method in the field and a recently published study by Osmann, Selva and Feinstein [6] administered this scale to investigate symptomatology among journalists working during the COVID-19 pandemic, also reporting similar rates of PTSD symptoms as found in our overall sample. 

Contrary to Van Overmeire’s assertion that the journalists in our study failed to experience criterion A trauma, we confirm that all participants had experienced a criterion A trauma as identified by the Life Events Checklist (LEC) [7]. Journalists completed the LEC followed by the PCL, which instructed participants to think of their worst event and restate it before continuing, confirming whether it involved “actual or threatened death, serious injury or sexual violence.” [5]. The PCL also captures how the event was experienced, whether the participant witnessed it, whether they learned about it happening to a close friend or family member, or were repeatedly exposed to it as part of their job.

Participants’ index events were as follows: sudden or unexcepted death of someone close (19%), fire or explosion (14.3%), sudden or violent death (13.1%), severe human suffering (11.9%), combat or exposure to a warzone (10.7%), sexual assault (8.3%), life-threatening illness or injury (7.1%), physical assault (4.8%), transportation accident (3.6%), unwanted sexual experience (3.6%), assault with a weapon (2.4%), or childhood neglect (1.2%). Every journalist had experienced a criterion A event as defined by the DSM-5 and completed the PCL-5 in relation to this event. In our sample, the majority (62%) of journalists experienced occupational trauma through work on the scene (47.6%) or repeated exposure from the office (14.3%). Personal trauma was reported by 38% of journalists. There was no significant difference between the two groups on how the trauma was experienced.

Our study advances the work of Osmann et al. [6] by ensuring the PCL-5 was completed by journalists who had experienced a criterion A event. As such, we can be confident that the rates of PTSD symptoms reported in our study are a result of exposure to DSM-5-defined trauma.

Van Overmeire expressed concerns over how journalists in different roles (such as editors versus reporters) may have experienced traumatic events. The DSM-5 includes repeated exposure to occupational trauma as a criterion A event. As such, repeatedly reporting on or editing news related to human suffering associated with COVID-19 or the perceived personal threat of catching the virus associated with keywork would fit the definition of a criterion A event in the DSM-5. The majority of editors in our sample (*n* = 25) identified occupational trauma as their index trauma with a minority (*n* = 6) having reported trauma experienced in their personal lives. 

Van Overmeire indicates concern over our reference to subthreshold PTSD. The DSM-5 includes other- and unspecified trauma and stressor-related disorders to capture subthreshold PTSD symptoms. However, the DSM-5 fails to specify number or type of symptoms associated with subthreshold PTSD, so we turned to published research as a guide. We chose to adopt the Stein et al. [8] definition of subthreshold PTSD since it has a strong evidentiary base and can be applied to the DSM-5 definition of PTSD, as seen in Franklin et al. [9].

The issue of differing lifetime trauma exposure between journalists who did and did not repeatedly cover COVID-19 news is something that we controlled for in our analyses, first in our ANCOVA analysis and also in the regression analysis. Journalists who repeatedly covered COVID-19 had significantly greater PTSD symptoms than journalists who covered stories unrelated to COVID-19 even after controlling for lifetime trauma exposure. It should be noted that as time progressed during the pandemic, journalists repeatedly reporting on COVID-19, by virtue of their work, accrued greater exposure to lifetime trauma and as such, the fact that the two groups differ in lifetime exposure to trauma is unsurprising. 

We can confirm that the assumptions for each statistical test were met and included an examination of Q-Q plots for ANCOVAs, which were normally distributed, and calculation of variance inflation factor (VIF) values to examine multicollinearity between variables in the regression analysis. There were no VIF values above 1.8, which is well below the value of 10 [10], as well as the more recent cautionary value of 2.5 [11]. All tolerance values were below 1.

Van Overmeire queried the administration of the COVID-19 impact questionnaire, which assessed repeated reporting of COVID-19 and the effect the pandemic may have had on journalists (e.g., having had COVID-19, working longer hours etc.). This questionnaire was used to identify which category journalists fell into: repeatedly covering COVID-19 news or covering news unrelated to COVID-19. Therefore, those journalists in the ‘not repeatedly covering COVID-19’ group may have reported on COVID-19 once, and subsequently interviewed an individual with COVID-19 once. This accounts for the eight participants in the ‘not covering COVID-19 repeatedly’ group who indicated they interviewed someone with COVID-19. We refute the claim that this questionnaire of COVID-19 impact was biased towards one group. The questions were designed to apply to all journalists working during the pandemic, regardless of whether or not they repeatedly covered COVID-19 stories.

Our study adopted a cross-sectional design and as such no causal statements can be or were made. The regression analysis was conducted to investigate the relationship of common strategies for dealing with unwanted memories, a normal feature in the aftermath of trauma, to PTSD symptom severity. We do not see rumination as a symptom of PTSD. It is a thinking style often triggered by an unwanted memory and has previously been shown to predict PTSD in high-risk occupational groups [12]. 

We would like to thank Van Overmeire for bringing to our attention the typo in the manuscript, which makes the GHQ scores seem very high. The items were scored 1–4, instead of 0–3. The range of scores with the 0–3 scoring is 12–48, and as such, the mean scores are in the mid-range. The 1–4 scoring did not affect the subsequent analysis. The 0–3 scoring was published with the original manuscript as a Correction. 

In efforts to conduct the most rigorous analysis of published research, there is risk that one loses sight of shared intentions: to advance science in a field capable of reducing human suffering. Nothing underscores the value of this work more clearly than the voices of journalists who worked tirelessly to report on COVID-19 at the peak of the pandemic when there was little understanding and immense fear. A BBC journalist tells us: *“It's almost like you're sort of being attacked by the news because you know, it's our job to be across everything… so I find myself being really on edge, quite a lot. When you finish, go home for the day, you're still kind of on a state of high alert. The offshoot of that is that it's quite hard to switch off, the end of the day. It can affect your sleep, your mood, your mental health.”*

Our research reports rates of PTSD symptoms among journalists working during the pandemic that are consistent with published studies and which advance prior research: we assess for exposure to DSM-5-defined criterion A events and subsequent PTSD symptom severity. Our data demonstrate that exposure to trauma among journalists is high with PTSD symptoms being highest among journalists repeatedly covering COVID-19. We suggest the high rates of PTSD symptoms may relate to exposure to COVID-19 trauma, may be related to prior trauma, which could increase vulnerability to PTSD symptoms during pandemic working, and reflect the persistence of PTSD symptoms associated with prior trauma or the cumulative effect of trauma exposure. Drawing on our data, we offer evidence-based recommendations for future interventions.
